# Interactive impacts of social deprivation and intranasal oxytocin administration on oxytocin receptor density in prairie vole brains

**DOI:** 10.3389/fnbeh.2026.1772423

**Published:** 2026-06-10

**Authors:** Susanna Zheng, George S. Prounis, Alexander G. Ophir

**Affiliations:** Department of Psychology, Cornell University, Ithaca, NY, United States

**Keywords:** autoradiography, biparental care, early-life social experiences, intranasal oxytocin, oxytocin receptors, paternal absence, prairie voles, social isolation

## Abstract

**Introduction:**

Early-life social experiences have a profound impact on the development of the brain and behavior. Previous work has shown that prairie voles (*Microtus ochrogaster*) raised in a socially limited environment (raised by a single mother and isolated after weaning) engaged in more social behaviors compared to those raised in a socially enriched environment (raised biparentally and group-housed after weaning). Furthermore, intranasal oxytocin has been therapeutically used in children, yet little is known about how this might affect brain development. Oxytocin is a crucial neuropeptide involved in social behavior and cognition, and variation in parental care and early social experiences has the potential to alter oxytocin receptors (OTR) throughout the brain. However, few studies have asked how different social experiences interact to alter brain phenotype.

**Methods:**

In this study, male prairie voles were raised in either a socially limited condition (raised by a single mother and isolated after weaning) or raised in a socially enriched condition (raised biparentally and group housed after weaning). Subjects in each condition were also administered either intranasal oxytocin or saline (PND 21-42). Finally, brains were collected, and we used autoradiography to quantify OTR throughout the brain of these animals.

**Results:**

It showed that much of the forebrain did not show differences in OTR density as a result of either manipulation, reinforcing the idea that OTR phenotype is generally resistant to external forces. However, we found two important exceptions to this theme in the prefrontal cortex and the lateral septum – two areas of the brain that are critical to social behavior in general and prairie vole pair bonding in particular. Specifically, socially limited animals had significantly more OTR in the prefrontal cortex compared to socially enriched animals, and subjects administered intranasal oxytocin during adolescent development expressed more OTR in the lateral septum compared those treated with saline.

**Discussion:**

Although the impacts of external sources on OTR density appear to be restricted to only a few locations in the brain, these brain regions are central to social functioning, indicating limited but potentially significant impacts on social behaviors are plausible.

## Introduction

Neural signaling between oxytocin (OT) and its receptor (OTR) is a fundamental regulator of complex social behaviors, particularly in mammals (Caldwell and Albers, 2016; Feldman, 2015a; Feldman, 2015b; Freeman et al., 2024; Neumann, 2008; Rilling and Young, 2014; Walum and Young, 2018). Its widespread influence highlights a critical role in coordinating social behavior and the functional “social brain” (Prounis and Ophir, 2020). Indeed, the influence of oxytocin has profound impacts on several interrelated but often discrete social behaviors.

Oxytocin is best known for facilitating social bonds. Classic work in prairie voles showed that central OT administration promotes partner preference formation (Williams et al., 1994), whereas disrupting OT signaling impairs its formation or maintenance (Insel and Hulihan, 1995; Liu and Wang, 2003; Young and Wang, 2004). Moreover, OTR density throughout the brain contributes to species-specific social organization (Beery et al., 2008; Campbell et al., 2009; Freeman et al., 2020; Insel et al., 1991; Insel and Shapiro, 1992; Turner et al., 2010). In particular, OTR in the nucleus accumbens (NAc) is essential for partner preference formation and mating-related reward (Aragona et al., 2003; Forero et al., 2023; Liu and Wang, 2003; Resendez et al., 2013; Young and Wang, 2004). OTR density in the NAc is typically greater in monogamous species (Freeman et al., 2020; Insel and Shapiro, 1992; Ross et al., 2009) and in pair-bonded males compared to single males in field conditions (Ophir et al., 2012), reinforcing the idea that it functions to facilitate partner-associated reward (Young and Wang, 2004; Young et al., 2005). OTR binding in the prefrontal cortex (PFC) is also necessary for partner preference formation (Young et al., 2001), making the PFC an important component of the “pair bonding neural circuit” (Johnson et al., 2017; Young and Wang, 2004; Young et al., 2005).

Beyond pair bonding, oxytocin regulates parental care. It is classically associated with maternal functions such as labor, lactation, and maternal bonding (Gimpl and Fahrenholz, 2001), but its influence on parental care extends across species (Chokchaloemwong et al., 2013; Dan et al., 2024; DeAngelis et al., 2020; Dulac et al., 2014; Mennigen et al., 2022; Numan, 2020). In rodents, hypothalamic OT is central to maternal behaviors like pup retrieval and huddling (Carcea et al., 2021), and OTR density correlates with variation in maternal care (Champagne et al., 2001). Oxytocin also influences non-parental caregiving, as OTR density in the lateral septum (LS) and striatum correlates with alloparental care (Olazábal and Young, 2006).

Early work on oxytocin emphasized its role in social recognition and other forms of learning and memory (McEwen, 2004; Ophir, 2017). OTR density in regions such as the LS, medial amygdala (MeA), hippocampus (HPC), and septohippocampal nucleus (SHi) is associated with learning, memory, and reproductive decision-making (Ophir, 2017; Ophir et al., 2012). For instance, OT signaling in the LS is critical for social recognition, with dose-dependent effects: low doses facilitate memory, whereas high doses impair it (Popik et al., 1992a,b). Lower LS OTR density is also linked to reduced investigation of females (Ophir et al., 2009).

Taken together, the oxytocin signaling system has great potential to serve a major role in coordinating social behavior across the brain. Furthermore, the oxytocin system is highly plastic and sensitive to environmental influences (Ahern and Young, 2009; Bales et al., 2011; Bales and Perkeybile, 2012; Bosch et al., 2018; Carter et al., 2009; Danoff et al., 2023; Dumais and Veenema, 2016; Hiura et al., 2024; Hiura and Ophir, 2018; Kelly et al., 2018; Rogers and Bales, 2020; Rogers et al., 2021; Tabbaa et al., 2017), making it well-suited for studying how early experiences shape neural development. It is well-established that early attachment quality predicts later social and emotional outcomes in humans (Bowlby, 1988, 1992; Salter Ainsworth et al., 1978; Sroufe, 2005). In rodents, variation in parental care influences OTR distribution and behavior. High-contact maternal care increases OTR density in regions involved in social behavior and is transmitted across generations (Champagne et al., 2001; Francis et al., 2000, 2002). In contrast, maternal separation increases anxiety, aggression, and social deficits, alongside region-specific changes in OTR binding and gene expression (Kalinichev et al., 2002; Lukas et al., 2010; Veenema, 2009; Wei et al., 2020). Similarly, prairie voles receiving high parental care show increased prosocial behaviors and higher OTR density in the BNST (Arias Del Razo and Bales, 2016; Perkeybile et al., 2013, 2015). Paternal absence delays partner preference formation and reduces OTR density in the central amygdala (Ahern and Young, 2009; Rogers et al., 2021), and also affects later parental behavior (Ahern et al., 2011).

Post-weaning experiences also shape the social brain. Juvenile social isolation increases anxiety, reduces affiliation, and promotes depression-like behaviors (Donovan et al., 2020; Grippo et al., 2007a,b). It also alters oxytocin-related physiology and impairs partner preference formation, particularly in females (Grippo et al., 2007b; Barrett et al., 2015). However, high OTR density in the NAc can buffer against these effects (Barrett et al., 2015), consistent with evidence that oxytocin mediates social buffering of stress (Burkett et al., 2016; Chun et al., 2022; File and Peet, 1980; Hirota et al., 2020; Sailer et al., 2022; Smith and Wang, 2014). Social enrichment after weaning increases OTR density in forebrain regions such as the LS and NAc (Prounis et al., 2018).

Experimental manipulation of oxytocin has traditionally relied on invasive methods (Donaldson and Young, 2008; Johnson and Young, 2015), but intranasal oxytocin (IN-OT) offers a non-invasive alternative that elevates central oxytocin levels (Neumann et al., 2013). IN-OT has shown therapeutic promise, including improving emotion recognition in individuals with autism (Guastella et al., 2010; Kong et al., 2023). It also affects social behavior in prairie voles, with dose-dependent effects on social contact and partner preference (Bales et al., 2013), and it enhances paternal care in both voles and humans (Weisman et al., 2012). Notably, IN-OT does not broadly affect all oxytocin-related behaviors, as it does not alter spatial memory performance (Finton and Ophir, 2022), though it modulates activity in brain regions involved in social and emotional processing (Domes et al., 2007; Xin et al., 2020; Koch et al., 2015; Kenkel et al., 2019; Sabihi et al., 2017).

Given its translational potential, understanding the long-term developmental effects of IN-OT is critical. Social behavior and neural phenotypes are highly sensitive to early-life experiences, which in natural settings often occur in combination. Thus, studying IN-OT within “multi-hit” developmental contexts is essential for understanding how complex social environments shape brain development.

Evidence suggests that adolescent IN-OT exposure can produce lasting effects. Prounis and Ophir (2019) examined how early social environments modulate these effects in prairie voles. In nature, some voles are raised without fathers (Getz et al., 1993), and some experience social isolation during dispersal (McGuire et al., 1993). Modeling this variation, Prounis et al. (2015) compared “socially limited” animals (paternal deprivation and isolation) with “socially enriched” animals (biparental care and group housing). Socially limited animals showed deficits in social recognition and altered OTR distribution, including increased LS OTR (Prounis et al., 2015). Follow-up work found that chronic IN-OT enhanced alloparental care and reduced aggression toward pups, but only in socially limited males, with effects persisting into adulthood (Prounis and Ophir, 2019), suggesting long-term organizational changes in social behavior.

Given the strong interaction between early experiences and the oxytocin system, it is essential to determine how IN-OT influences OTR development across the brain. To our knowledge, no studies have systematically examined how combinations of early social environments and IN-OT exposure shape neural development. Here, we investigated how socially enriched and socially limited rearing conditions interact with intranasal oxytocin to influence oxytocin receptor density in the brain.

## Materials and methods

### Animals

Prairie voles used in this study were offspring of breeding pairs created from of wild-caught prairie voles from Champaign, IL, USA. All animals were housed in standard polycarbonate rodent cages (29 × 18 × 13 cm) lined with Sani-chip bedding and provided nesting material, and were kept on a 14:10 light-dark cycle. Animals were also provided a diet of rodent chow (Laboratory Rodent Diet 5001, Lab Diet, St Louis, MO, United States) and water ad libitum. Colony and behavior observation rooms were kept on a 14L:10D light cycle (lights on at 0700) with room temperatures maintained at 20 °C + 2. Sex was determined based on external genitalia. All procedures were approved by the Institutional Care and Use Committee of Cornell University. All the subjects in this study were subjects used in Prounis and Ophir (2019).

### Early life manipulations

**FIGURE 1:**
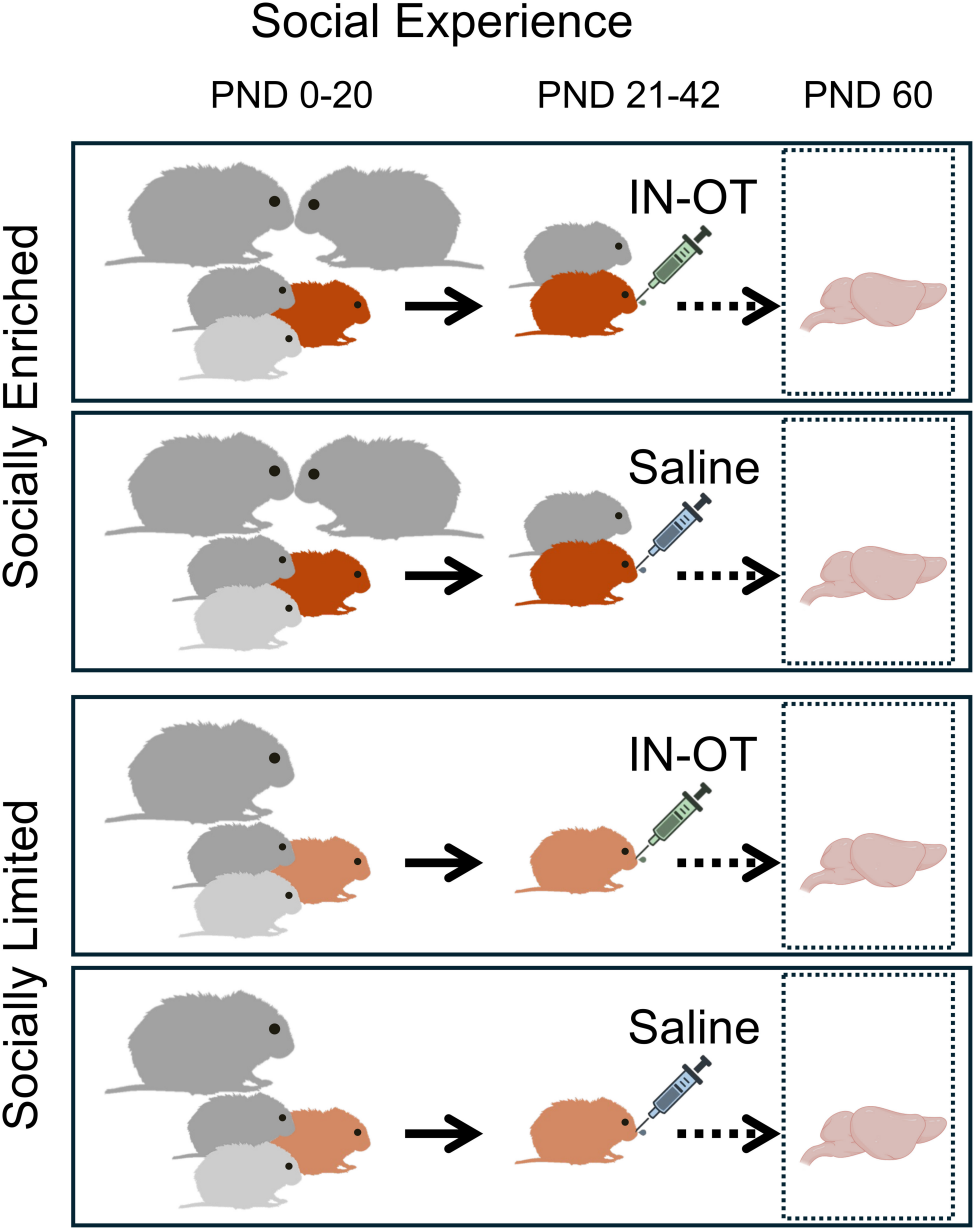
Experimental timeline. Male prairie voles were either raised in a socially enriched environment (biparentally raised and then group housed after weaning; represented by dark orange) or socially limited environment (raised by single mother and then isolated after weaning; represented by light orange). Half of the animals experiencing each social-experience regimen were either administered intranasal oxytocin (IN-OT; green) or saline (blue) during the adolescent period (PND 21-42). Brains were extracted at adulthood (PND 60) to perform oxytocin receptor autoradiography.

Sexually mature adult (>60 days old) prairie voles were paired together and allowed to breed freely. Breeders were checked for litters about 20 days post-pairing (gestation lasts approximately 21 days). A male from the first litter of each breeder pair served as the subjects for the experiment. At birth, male pups were assigned to one of the two social experience conditions, which spanned pre-weaning and post-weaning development: “socially enriched” or “socially limited.” In the socially enriched condition, male pups were reared biparentally until weaning [postnatal day (PND) 20] and then group-housed with a male sibling. In the socially limited condition, the father was removed at PND 0 and pups were reared by only their mother. At weaning, the male subjects were housed alone. See Figure 1 for experimental timeline. We use the terms “socially enriched” and “socially limited” to label the treatments in an overly simplistic way to convey that the social experiences we created incorporated social opportunities with more or fewer individuals, relative to each other. We do not intend for these terms to convey preconceived notions that one condition is “better” or more adaptive than the other.

### Intranasal oxytocin treatments

After weaning, subjects received daily intranasal treatments of oxytocin (0.8 IU/kg) or saline control between 08:00 and 12:00 h for 3 weeks, spanning adolescence (PND 21–42; Figure 1). We applied a total of 25 μl of saline or OT from a pipette tip around the nasal cavity while the subject was scuffed and held belly-up. To administer OT or the saline control, we alternated sides of the nose, so that 12.5 μl was applied to each nostril. This ensured the solutions were fully inhaled. We note that subjects were tested on a series of juvenile affiliation tests at PND 2—42, alloparent care tests at PND 43 and 58, and a partner preference test at PND 60; results of these tests are reported in Prounis and Ophir (2019).

### Tissue collection

Animals were euthanized via CO2 inhalation when they were 60 days old, and brains were immediately removed and flash frozen on powdered dry ice. Whole brains were stored at −80 °C prior to sectioning. To prepare brains for autoradiography (see below) each brain was coronally sectioned at 20 μm thick using a Leica cryostat (CM1950, Leica Biosystems, Nussloch, Germany) set at −20 °C. We mounted 4 sets of sectioned brains, with 100 μm intervals between each section, on Superfrost Plus Microscope sides (Fisher Scientific, Pittsburg, PA, United States). Sectioning began at the level of the olfactory bulbs and spanned to the start of the cerebellum. Sectioned tissue was stored in microscope slide boxes (Fisher Scientific, Pittsburg, PA, United States) at −80 °C until the autoradiography procedure.

### Autoradiography

One set of sectioned brain tissue was thawed to visualize OTR density across the forebrain using the ^125^I-labeled radioligand (ornithine vasotocin analogue ([125I]-OVTA); NEX 254, PerkinElmer; Waltham, MA, United States). Competitive binding studies for this ligand have consistently demonstrated high selectivity (see Taylor et al., 2020). To process tissues, we lightly fixed sections on slides in 0.1% paraformaldehyde for 2 min, washed them two times in 1X Tris for 10 min, and incubated them with 40pM [125I]-OVTA for 90 min. Next, we washed slides in a series of 5 min baths of 1X Tris with MgCl_2_ followed by a final wash in 1X Tris with MgCl_2_ for 30 min, briefly dipped them in filtered water, and then rapidly air-dried them.

Once dried, slides containing the radiolabeled tissue were placed in light-proof autoradiography cassettes and exposed to film (Biomax MR, GE Healthcare) for 4 days. We placed ^125^I labeled radiographic standards (American Radiolabeled Chemicals; St Louis, MO) in each cassette to allow for conversion of film optical density to receptor density (see below). After the film was developed, we digitized films on a Microtek ArtixScan M1 (Microtek, Santa Fe Springs, CA) and measured optical densities using NIH ImageJ Software (NIH, Bethesda, MD).

The relative density of ligand binding was assessed by inferring that receptor density relates to the optical density of exposed film, and in this way optical density measurements serve as a proxy for receptor density. Thus, we calculated receptor density by first converting optical density to disintegrations per minute (dpm) adjusted for tissue equivalence (TE; for 1 mg in the rat brain). We next used a log function to fit curves generated by the radiographic standards and extrapolated OTR density based on these standard curves for each film (see Ophir et al., 2013).

**FIGURE 2:**
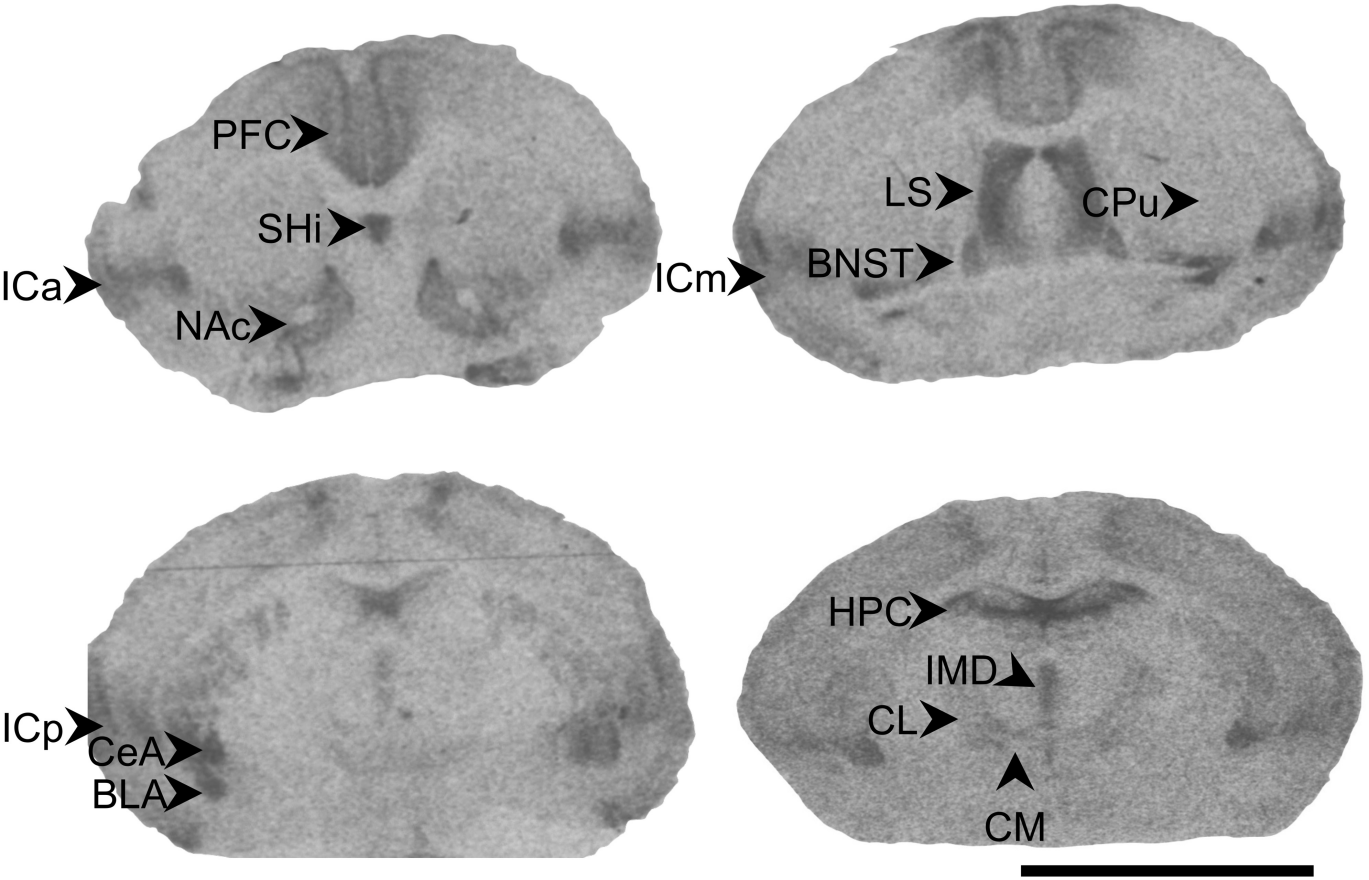
Oxytocin receptors (OTR) autoradiograms. Autoradiograms of OTR ([125-I]-OVTA specific binding) across the forebrain. Arrows indicate the prefrontal cortex (PFC), anterior insular cortex (ICa), nucleus accumbens (NAc), septohippocampal nucleus (SHi), lateral septum (LS), bed nucleus of the stria terminalis (BNST), caudate-putamen (CPu), medial insular cortex (ICm), central amygdala (CeA), basolateral amygdala (BLA), hippocampus (HPC), posterior insular cortex (ICp), the intermedial dorsal thalamic nucleus (IMD), centromedial thalamic nucleus (CM), and centrolateral thalamic nucleus (CL). Scale bar 5 mm.

We measured optical density for each structure of interest three times (once on a series of three brain sections, bilaterally). We also measured nonspecific binding on each section by measuring the background levels of cortex (bilaterally) in areas that do not express OTR on each of the same sections measured. The values for each structure were averaged, converted to dpm/mg TE (as just described), and adjusted to represent specific binding by subtracting nonspecific binding from total binding for each area. We measured OTR in the prefrontal cortex (PFC), anterior insular cortex (ICa), nucleus accumbens (NAc), septohippocampal nucleus (SHi), lateral septum (LS), bed nucleus of the stria terminalis (BNST), caudate-putamen (CPu, i.e., the striatum), medial insular cortex (ICm), central amygdala (CeA), basolateral amygdala (BLA), hippocampus (HPC), posterior insular cortex (ICp), and the intermedial dorsal thalamic nucleus (IMD), centromedial thalamic nucleus (CM) and centrolateral thalamic nucleus (CL), collectively referred to here as the thalamus (Thal) for simplicity. Representative audioradiograms are presented in Figure 2. These images were manipulated using Adobe Photoshop (version 26.7.0) for brightness (-25), contrast (-50) and curves (input: 200, output: 16), and then converted to gray scale from RGB mode. Film backgrounds were removed.

### Experimental design and statistical analysis

Our experiment consisted of a two-factor design with two levels for each factor: Social Experience (enriched or limited) and Intranasal Treatment (OT or Saline). Sample sizes for each group were: Socially Enriched + Saline, *N* = 11; Socially Enriched + OT, *N* = 10; Socially Limited + Saline, *N* = 12; Socially Limited + OT, *N* = 12. However, sections of the SHi for one individual were lost due to tissue damage, reducing the Socially Enriched + Saline group to *N* = 11.

Mean OTR density was analyzed using a full factorial two-factor ANOVA design with the Prism statistical package (v 10.6.1; GraphPad). We included Social Experience (limited or enriched) and Intranasal Treatment (oxytocin or saline) as fixed factors. Fisher’s LSD post-hoc comparisons were run when ANOVA detected a significant effect. An independent model was run for each brain region of interest, as is the convention for such analyses (e.g., Beery et al., 2008; Duchemin et al., 2017; Freeman et al., 2014, 2019; Hammock and Levitt, 2013; Hiura and Ophir, 2018; Ophir et al., 2013; Prounis et al., 2018; Smith et al., 2017; Sommer and Caldwell, 2025), because each region was considered an independent a priori comparison. For all tests, an alpha cutoff of <0.05 was used to determine statistical significance.

## Results

Notably, most brain regions that we examined demonstrated robust and consistent OTR density across conditions. Indeed, neither Social Experience nor Intranasal Treatment impacted OTR density in the NAc, ICa, SHi, BNST, CPu, ICm, CeA, BLA, HPC, ICp, or Thal (see Table 1). Likewise, the interaction between Social Experience and Intranasal Treatment was not significant in these regions of interest. Taken together, these results provide evidence indicating that OTR density is relatively unaffected by the combination of the specific forms of early-life social experiences our subjects experienced and that IN-OT does not appear to have wide-ranging impacts on the development of OTR in the forebrain of developing adolescent prairie voles. However, OTR density was impacted by Social Experience in the prefrontal cortex and Intranasal Treatment in the lateral septum (see below).

**TABLE 1:** Statistical results for our social experience by intranasal treatment contrasts examining oxytocin receptors (OTR) density across the forebrain.

Brain region	Social experience		Intranasal treatment		Interaction: SE × IT	
	*F*_(1,41)_	*P*	*F*_(1,41)_	*P*	*F*_(1,41)_	*P*
PFC	**6.14**	**0.02**	1.58	0.22	1.10	0.30
NAc	0.77	0.39	0.48	0.49	0.10	0.74
ICa	0.04	0.85	0.07	0.80	0.65	0.43
SHi*	0.04	0.84	0.31	0.58	1.31	0.26
LS	0.38	0.54	**5.94**	**0.02**	1.53	0.22
BNST	0.07	0.78	0.14	0.71	0.54	0.47
CPu	0.04	0.83	0.03	0.87	1.18	0.28
ICm	0.75	0.39	2.61	0.11	2.44	0.13
CeA	0.17	0.68	0.28	0.60	2.85	0.10
BLA	0.07	0.79	0.45	0.51	0.42	0.52
HPC	0.25	0.62	0.60	0.44	0.14	0.71
ICp	0.01	0.93	1.65	0.21	0.03	0.87
Thal	0.13	0.72	0.10	0.75	1.66	0.21

Significant differences are in bold. PFC, prefrontal cortex; ICa, anterior insular cortex; NAc, nucleus accumbens; SHi, septohippocampal nucleus; LS, lateral septum; BNST, bed nucleus of the stria terminalis; CPu, caudate-putamen; ICm, medial insular cortex; CeA, central amygdala; BLA, basolateral amygdala; HPC, hippocampus; ICp, posterior insular cortex; Thal, thalamus. *For the SHi, the degrees of freedom were *F*_(1, 40)_ because sections for one individual were lost due to tissue damage.

### Prefrontal cortex

**FIGURE 3:**
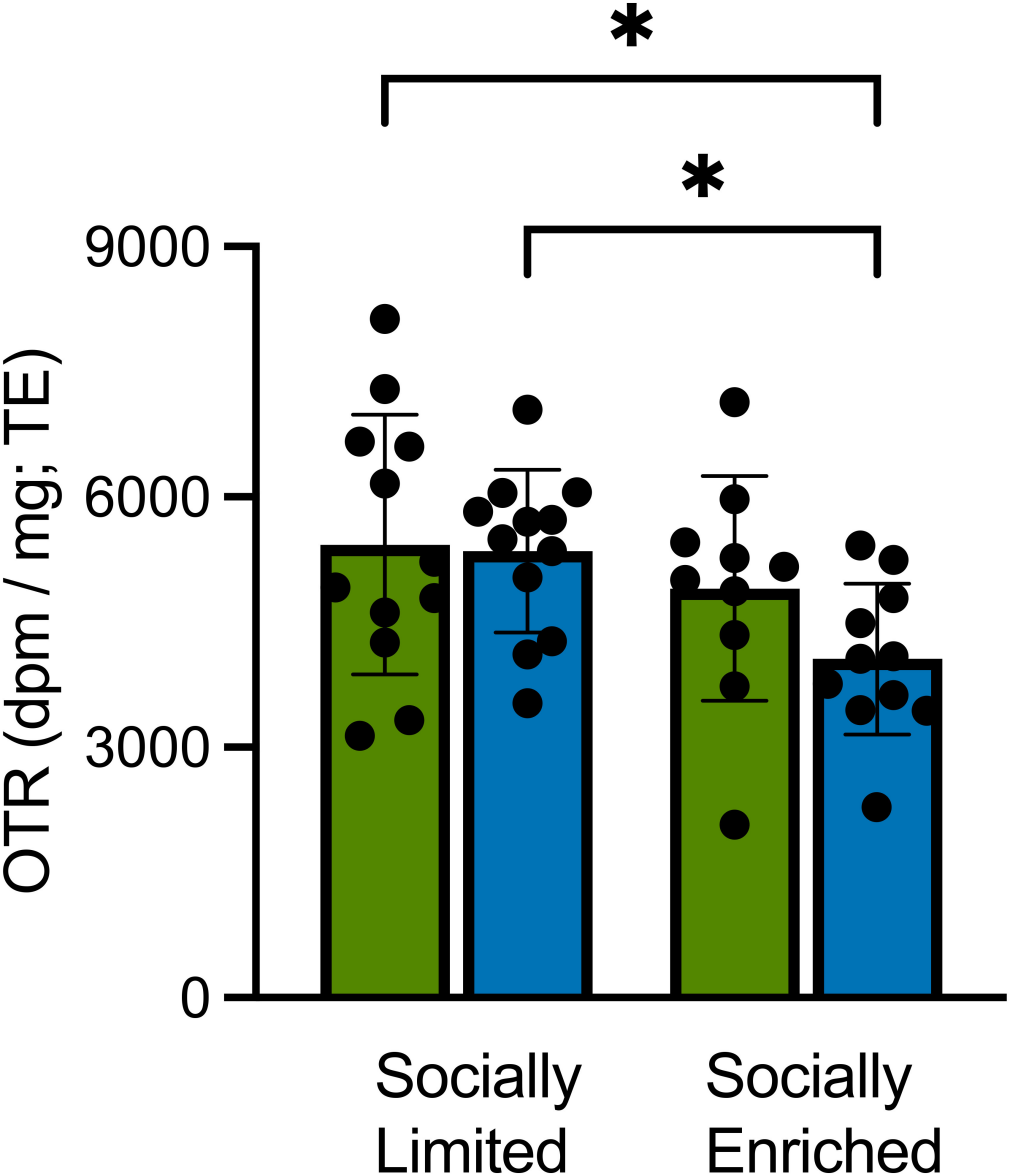
Prefrontal cortex oxytocin receptors (OTR) density. Mean (+SE) oxytocin receptor density (dpm/mg TE). Green bars represent IN-OT treated animals. Blue bars represent saline treated animals. Dots represent individual animals. *p < 0.05.

We found a significant main effect of Social Experience [F_(1,41)_ = 6.14, *p* = 0.02; Figure 3] in the prefrontal cortex, with socially limited individuals exhibiting greater OTR densities than socially enriched individuals. However, intranasal treatment did not impact OTR density in the PFC [F_(1, 41)_ = 1.58, *p* = 0.22], and the interaction between social experience and intranasal treatment was not significant [F_(1, 41)_ = 1.095, *p* = 0.30]. Our post hoc analyses indicated that socially limited males receiving IN-OT or saline did not differ in PFC OTR density (Fisher’s LSD: LS mean difference = 77.8; *p* = 0.88), and socially enriched individuals receiving IN-OT or saline did not differ (LS mean difference = 844.7; *p* = 0.12). Notably, socially enriched and socially limited males that received saline significantly differed (LS mean difference = 1291.0; *p* = 0.02), with socially limited males exhibiting greater OTR density. Socially enriched males receiving intranasal saline also showed greater OTR density compared to socially limited males treated with IN-OT (LS mean difference = 1369.0; *p* = 0.01). No other post hoc contrasts were significantly different (all LS mean differences < 524.4; *p* > 0.32). Aligning with other studies demonstrating the susceptibility of OTR phenotype to social experience (see below), these results suggest that a dual-hit of reduced social interactions in early-life may increase OTR density in the PFC. We also note that OTR in the PFC did not significantly correlate with behaviors measured in alloparental care tests (huddling or attacks) recorded at PND 43 or 58 or the proportion of time subjects spent with a partner in a partner preference test (see Supplementary material; Prounis and Ophir, 2019).

### Lateral septum

**FIGURE 4:**
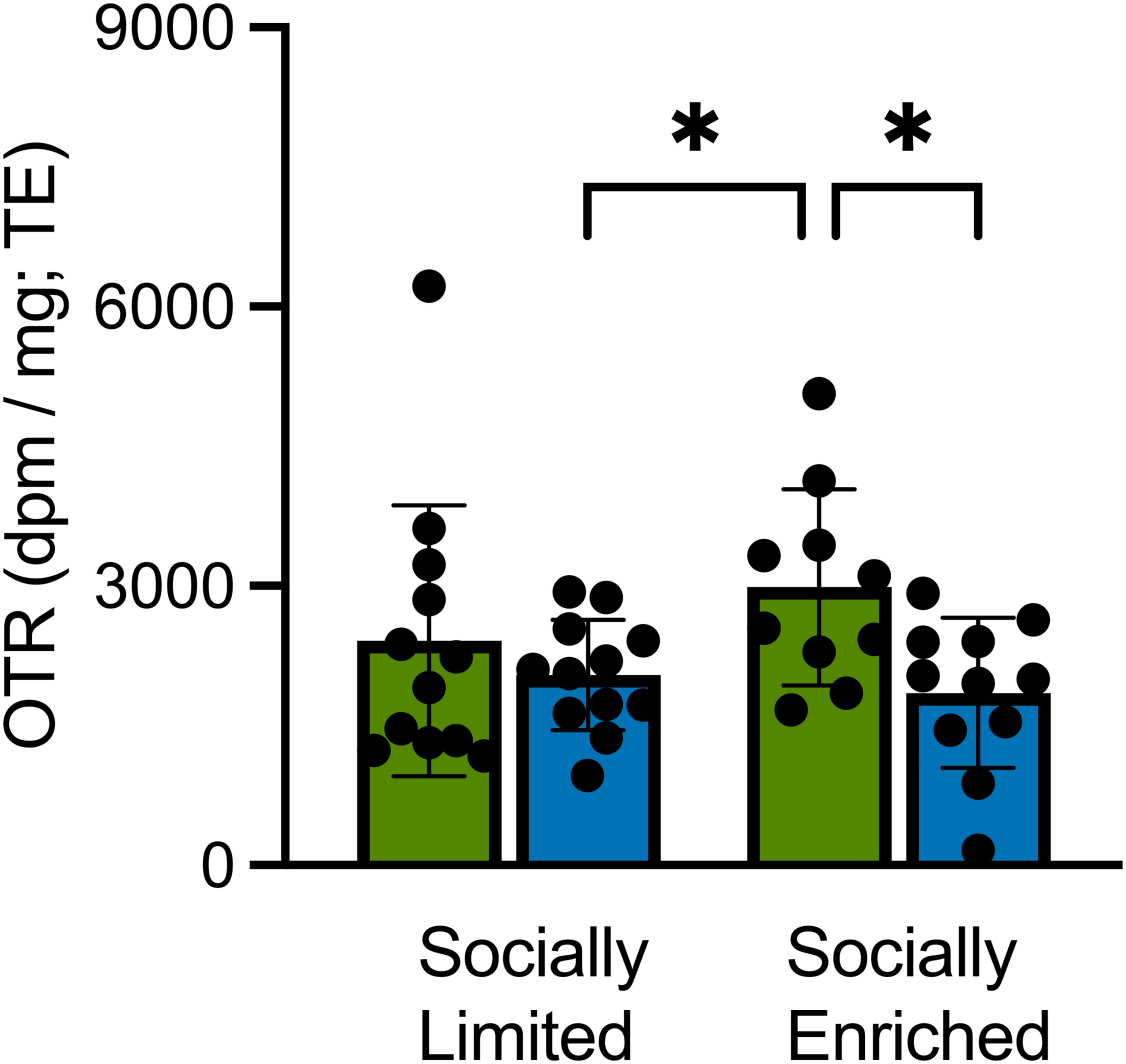
Lateral septum oxytocin receptors (OTR) density. Mean (+SE) oxytocin receptor density (dpm/mg TE). Green bars represent IN-OT treated animals. Blue bars represent saline treated animals. Dots represent individual animals. *p < 0.05.

Although Social Experience did not impact LS OTR density in the lateral septum [F_(1, 41)_ = 0.38, *p* = 0.54], we found a significant main effect of Intranasal Treatment [F_(1, 41)_ = 5.94, *p* = 0.02; Figure 4], where IN-OT enhanced OTR density in the LS. Social Experience and Intranasal Treatment did not significantly interact [F_(1, 41)_ = 1.53, *p* = 0.22]. Our *post hoc* analyses showed that the main effect of IN-OT treatment was primarily driven by its effect in socially enriched subjects. Specifically, socially enriched IN-OT treated subjects showed significantly greater OTR density in the LS socially enriched saline treated subjects (LS mean difference = 1134.0; *p* = 0.02), whereas the post hoc difference between socially limited IN-OT treated subjects and socially limited saline treated subjects fell short of significance (LS mean difference = 370.7; *p* = 0.38). Socially enriched males receiving IN-OT also showed greater OTR density than socially limited males receiving saline (LS difference −942.1; *p* = 0.04). IN-OT treatment between socially enriched and socially limited males (LS mean difference = −571.5; *p* = 0.20) and saline treatment between socially enriched and socially limited males (LS mean difference = 191.5; *p* = 0.66) did not differ in OTR density. Thus, chronic IN-OT delivered over the course of adolescence has the potential to alter OTR density in a central regulator of the social brain (see below). We also note that OTR in the LS did not significantly correlate with behaviors measured in alloparental care tests (huddling or attacks) recorded at PND 43 or 58 or the proportion of time subjects spent with a partner in a partner preference test (see Supplementary material; Prounis and Ophir, 2019).

## Discussion

We investigated the consequences of the interactions between early-life social experiences and chronic IN-OT administration on OTR density throughout the brain of male prairie voles. In doing so, we employed a biological meaningful method of stacking a combination of two common social experiences (experience with two parents or only one during perinatal development and then experience with social companionship or isolation during adolescence, respectively) to produce animals that had relatively enriched or relatively limited social exposure during their development. We also exposed adolescent animals to intranasal oxytocin (or a saline control) to determine if exogenous non-invasive OT exposure might compensate for the reduced social experience on the brain, and to determine if chronic exposure to OT during adolescent development might impact the OTR neural phenotype. We have reported previously that such treatment has profound impacts on prosocial behaviors, including juvenile social affiliation and alloparental care (Prounis and Ophir, 2019). Although not widespread, the variation produced by these experimental manipulations revealed important insights into the susceptibility of OT-OTR functioning to external forces. By characterizing how OTR expression is modified by the combination of paternal absence and post-weaning social isolation, or exogenous oxytocin exposure, we aimed to elucidate physiological pathways potentially linking early developmental context to long-term modulation of the neural mechanisms underlying social behavior.

Developmental differences in OTR between juveniles and adults are quite common in rats (Smith et al., 2017). In contrast, Prounis et al. (2018) showed that standard housed male prairie voles show very little change in OTR density over development across the forebrain. Notably, at least four brain regions that express OTR demonstrated significant increases in OTR density over development (PND 6–12, and PND 15–21) and into adulthood (PND 60) among male prairie voles, including the PFC, CPu, NAc and CeA. Nevertheless, this collection of brain areas that demonstrated OTR plasticity over development represents a profound minority of prairie vole brain areas known to express OTR. Furthermore, most of the change noted in these structures tended to occur before reaching PND15 (Prounis et al., 2018). Consistent with this relative robustness of OTR over development, we found that the combinatorial impact of social experiences and IN-OT administration did not impact OTR density in most of the forebrain, indicating that the male OTR phenotype is indeed robust to external forces.

It is important to note that other studies have found that different degrees of natural OTR density variation across brain regions, that some of this variation predicts behavioral variation, and that some social experiences induce differences in OTR phenotype. For example, prairie voles living in outdoor field conditions show tremendous variation in OTR in some regions of the brain, including the SHi, HPC, and Thal, but relatively low levels of natural variation in others, such as the PFC, NAc, and LS (Ophir et al., 2012). Interestingly despite relatively low levels of naturally occurring variation, differences in NAc OTR density predict which animals are monogamously paired (high) or remain unpaired (low), and differences in ICp OTR density predicted which animals bred successfully (high) or did not breed (low) (Ophir et al., 2012). Thus, the potential for experience to modify OTR density might depend on the structure and the specific degree to which that brain region has the capacity to vary. And yet, the two structures in which we found OTR differences in the current study (PFC and LS) show relatively low natural variation in OTR (Ophir et al., 2012). This indicates that although high degrees of natural variation could serve as a foundation upon which experiences could modify receptor expression, the areas of the brain with less variation might be a more fruitful place to look for environmental/experiential impacts of OTR modification. On the other hand, such variation should not be dismissed, and other sources (potentially genetic; e.g., King et al., 2016; Lee et al., 2025) might be better pathways to explain the structural-functional relationships of OTR in other regions of consequence.

Beyond the natural variation in OTR, some studies have shown that paternal deprivation and social isolation after weaning increases OTR density in the NAc (Hiura and Ophir, 2018; Prounis et al., 2015) and animals reared without fathers demonstrate greater OTR density in the CeA (Ahern and Young, 2009). Oddly, we did not find differences in the NAc or CeA. It is challenging to determine why our study demonstrated that OTR density was either unchanged in so much of the forebrain or why our study was inconsistent with the few differences reported in other studies. We suspect these inconsistent results largely come down to differences in the experiences that our animals received compared to those in other studies, whether it was the specific form of early-life social experiences that animals had, the combinatorial approach of multiple types of early-life social experiences animals from this study received, and/or the administration of IN-OT that was overlaid on these experiences. If this interpretation is true, these differences between studies underscore the complex relationship within and between different types of social experiences and the multiple ways in which this can potentially impact the developing brain.

Nonetheless, we did find some notable differences in the prefrontal cortex and the lateral septum, and exploring these results might provide some insights into the ways the external world can impact OTR phenotype. Firstly, our data indicated that socially limited voles (paternal deprivation + isolation group) had significantly higher OTR density in the PFC. The PFC plays a key role in executive function, emotion regulation, and social decision-making (Dalley et al., 2004; Miller and Cohen, 2001). Several studies spanning multiple species have indicated that PFC OTR is open to the influence of social environments experienced in early-life (e.g., see below). Therefore, the sensitivity of OTR in the PFC to early-life social experiences may reflect the brain’s capacity to shape higher-order social cognition based on environmental input (Gee et al., 2013). Unfortunately, there does not appear to be a consistent pattern of how social experiences alter OTR within the PFC. For example, paternal deprivation appears to decrease oxytocin receptor mRNA and OTR of Mandarin voles (*Microtus mandarinus*) (Yuan et al., 2020). On the other hand, paternal deprivation was also found to increase OTR binding in the infralimbic cortex (a subregion of the medial PFC) of Mandarin voles (He et al., 2019). These kinds of inconsistencies seem to be relatively common and have been found elsewhere in the brain as well, for example the OTR mRNA in the MeA (Cao et al., 2014; Yuan et al., 2020). Like discussed above, these inconsistencies have also been attributed to methodological differences in the timing of early-life social experiences.

Furthermore, social deprivation can alter the oxytocin signaling system. Huang et al. (2021) found that socially isolated juvenile mice were more prosocial but exhibited reductions of OTR in the PFC. Contrary to this study, the prairie voles in the current study were also tested behaviorally and, although we found that the socially limited male voles engaged in more social contact as juveniles (Prounis and Ophir, 2019), we report here that these males exhibited greater PFC OTR density. Therefore, although such changes in PFC OTR might impact the sociability of individuals that experienced social isolation due to alterations of oxytocin sensitivity (Musardo et al., 2022), the form of experience-phenotype plasticity that alters oxytocin function seems to vary across experience and species. The effects we report suggest that PFC OTR could potentially function as a compensatory mechanism to counteract the social deficits of paternal deprivation and isolation. This interpretation is plausible when considering that the PFC regulates emotion and social decision making (Dalley et al., 2004; Miller and Cohen, 2001). However, stepping outside of our results and considering the multiple ways in which different experiences (and the timing of experiences) can impact PFC OTR, one must acknowledge that the only clear theme is that PFC OTR is malleable with respect to external social forces. Identifying ways to understand and even predict the direction of those impacts requires a more systematic and exhaustive exploration of this basic theme.

The lateral septum is a crucial component of the social decision-making network (O’Connell and Hofmann, 2011), functioning as a point of convergence between that network and the mesolimbic rewards system (Prounis and Ophir, 2020). Furthermore, the LS is a powerful locus for the neuromodulation of complex social behaviors, and it plays a prominent role in multiple behavioral and cognitive domains, including regulating social recognition and anxiety/stress responses, and contributing to pair bonding and social attachment (Bielsky and Young, 2004; McEwen, 2004; Neumann, 2008; Ophir, 2017; Ophir et al., 2009; Triana-Del Rio et al., 2022; Young and Wang, 2004). The LS is thought to integrate sensory input, assess its affective relevance, and subsequently direct motivated behaviors (Risold and Swanson, 1997; Sheehan et al., 2004; Swanson and Risold, 2000). OTR density generally remains stable across early postnatal development in both male and female prairie voles reared under standard conditions (Prounis et al., 2018; Rogers et al., 2021). However, in some instances, OTR density in the LS appears to be highly sensitive to the post-weaning social environment (Prounis et al., 2018). For example, males reared without fathers and subsequently housed in isolation (just as our socially limited animals were) demonstrated an apparent increase in LS OTR density, which was also associated with impaired social discrimination abilities (Prounis et al., 2015). In the current study, we surprisingly did not find a similar main effect of early social experience on OTR density, and instead we found that chronic IN-OT treatment during adolescence induced OTR density to significantly increase. This effect was particularly prominent among male prairie voles that were raised in the socially enriched group. Notably, intranasal oxytocin administration increases social interaction and empathy (Carter, 2014), behaviors with which the LS has been associated (Prounis and Ophir, 2020; Sheehan et al., 2004). Furthermore, IN-OT administration has been associated with profound changes in functional connectivity in mice. Specifically, acute administration (a one-time dose) enhanced localized functional connectivity within the limbic system and chronic administration (i.e., twice a day for 7 days) enhanced more widespread functional connectivity (Pagani et al., 2020), demonstrating that the extent of exposure to IN-OT does indeed have the potential to increase its footprint on the developing brain. The chronic administration of IN-OT in Pagani et al. (2020) was also associated with a paradoxical reduction in social interaction, indicating that extended IN-OT exposure reduced social behavior even though it boosted brain restructuring. Consistent with the ability of IN-OT to reduce prosocial behavior, Bales et al. (2013) demonstrated that chronic exposure to OT during the adolescent period (PND 21–42) impaired adult pair bonding in a dose-dependent fashion. In a follow up, and like our study, Guoynes et al. (2017) found that IN-OT administration to male prairie voles (across doses and on the same timescale) produced no changes in OTR in any brain region they examined, including the LS (however, they did not characterize the PFC). Thus, while most work indicates that IN-OT administration tends to reduce or impede prosocial behavior and it does impact brain function, rarely has IN-OT been linked with impacting OTR phenotype. Considering the far reach the LS has for impacting social behavior (Prounis and Ophir, 2020; Sheehan et al., 2004), this result serves as a warning that potentially profound consequences on social behavior could result from subtle effects within a limited but highly impactful region of the social brain.

The current study showed how the interaction of multiple early-life social experiences impact oxytocin receptors in the brain. Specifically, it examined how the interaction between individuals raised in socially limited conditions versus socially enriched conditions and intranasal oxytocin treatment shape oxytocin receptor phenotype. Taken together, our data show that the prefrontal cortex and lateral septum exhibited OTR plasticity resulting from manipulation of early-life social experiences or IN-OT exposure, respectively. We suspect that we were able to detect the differences reported here because of our approach to combine the study of IN-OT administration on the backdrop of complex manipulations of the social environment. Although this approach produced results that were inconsistent with other work at times, the majority of our results indicated that OTR is relatively robust and resistant to environmental factors. With a few important exceptions, this result is relatively consistent across studies. Perhaps most notably, exposure to exogenous oxytocin via intranasal delivery does indeed alter some aspects of OTR phenotype. However, it is reassuring to know that these effects are rather limited. Nevertheless, the regions of the brain in which our manipulations affected OTR raises the possibility that profound consequences on social behavior might be linked with important functional changes to the social brain. Indeed, continued work to uncover the extent to which IN-OT can impact brain-behavior dynamics is clearly warranted, especially if it is to be used for therapeutic purposes in children.

## Data Availability

The raw data supporting the conclusions of this article will be made available by the authors, without undue reservation.
