# Supplementary material for: Interactive impacts of social deprivation and intranasal oxytocin administration on oxytocin receptor density in prairie vole brains
Source: Front Behav Neurosci. 2026 Jun 10;20:1772423. doi: 10.3389/fnbeh.2026.1772423 (PMC13290860; doi:10.3389/fnbeh.2026.1772423)
Supplement: Supplementary file 1 [file Data_Sheet_1.docx]

**Supplementary Material for:**

*Zheng S, Prounis GS and Ophir AG (2026) Interactive impacts of social deprivation and intranasal oxytocin administration on oxytocin receptor density in prairie vole brains. Front. Behav. Neurosci. 20:1772423.*

The OTR receptor density data reported here were collected from animals for which we previously described how chronic administration of IN-OT and early-life social enrichment impacted several social behaviors. These were reported in Prounis & Ophir (2019).

In that study, we recorded behavior from subjects for Social Contact time with an unrelated unfamiliar juvenile conspecific at PND 22, 28, 35, and 42, and Huddling and Attacking unrelated pups in an alloparental care test at PND 43 and 58. We also assessed proportion of time in a partner preference test (PPT) spent with the partner at PND 60. Shortly thereafter, we collected brains as described in the main report.

Potential correlations between OTR density and these behaviors could provide some insight into the nature of the relationships between natural variation in region-specific OTR density and variation in these social behaviors. We note, however, that such analyses would not provide conclusive results beyond associations that may or may not be germane to the core question: Does early-life social experience and/or administration of intra-nasal OT impact brain phenotype? Therefore, we provide the correlations of these comparisons as supplementary material.

Furthermore, because these correlations amounted to exploratory analyses involving multiple comparisons, we restricted these analyses to the two brain regions for which we found OTR density differed in our original analyses: the PFC and the LS.

We note that we felt that correlating Social Contact time at PND 22-42 was unjustified because we the OTR phenotype that we observed at adulthood (PND 60, or later) would not represent the brain state earlier in development. We know that OTR density changes over development naturally, and OTR density is also particularly sensitive to social environment (as demonstrated by Prounis et al. 2018, JCN, for example). Similarly, comparing OTR phenotype at PND 60 with behaviors recorded from animals that were 43 days old or younger also raised concerns that such comparisons might not be justified. We note that PND 45 represents the sub-adult life history stage, a time when the prairie vole brain is still being organized (Finton et al. 2022, Hiura & Ophir 2018, Hiura et al. 2018, Hiura et al., 2024, Kelly et al. 2018a, Kelly et al. 2018b).

Thus, the only behaviors we felt were justified to examine were those recorded at PND 58 (the second alloparental care test) and PND 60 (the partner preference test). Nevertheless, for the purposes of providing the information to the interested reader, we provide results from both alloparental care tests (attacks and huddling) at PND 43 and PND 58, and the partner preference test at PND 60. However, we excluded the social contact correlations at PND 42 and younger.

**Prefrontal Cortex**: The results showed that OTR density in the PFC did not correlate with huddling or attacks in the alloparental care test at PND 43 (N = 43, r = -0.05, p = 0.75 and N = 44, r = 0.10, p = 0.51, respectively), or at PND 58 (N = 44, r = -0.043, p = 0.81 and N = 44, r = 0.04, p = 0.81, respectively), and did not correlate with the proportion of time with the partner in a PPT (N = 42, r = 0.29, p = 0.06). These values are not corrected for multiple comparisons.

**Lateral Septum**: Likewise, OTR density in the LS did not correlate with huddling or attacks in the alloparental care test at PND 43 (N = 43, r = -0.16, p = 0.30 and N = 44, r = -0.10, p = 0.51, respectively), or at PND 58 (N = 44, r = -0.23, p = 0.14 and N = 44, r = -0.09, p = 0.56, respectively), and did not correlate with the proportion of time with the partner in a PPT (N = 42, r = 0.12, p = 0.47). These values are not corrected for multiple comparisons.

**Figure SM1:** Prefrontal cortex OTR density (dpm/mg: disintegrations per minute per 1 mg of brain tissue equivalence) at PND 60 does not correlate with affiliative or aggressive behavior measured at PND 43, PND 58 or PND 60. Solid line represents the regression slope; Dashed lines represent 95% confidence bands.

**Figure SM2:** Lateral septum OTR density (dpm/mg: disintegrations per minute per 1 mg of brain tissue equivalence) at PND 60 does not correlate with affiliative or aggressive behavior measured at PND 43, PND 58 or PND 60. Solid line represents the regression slope; Dashed lines represent 95% confidence bands.

**References:**

Finton CJ, Kelly AK, Ophir AG (2022). Support for the parental practice hypothesis: Subadult prairie voles exhibit similar behavioral and neural profiles when alloparenting kin and non-kin. *Behavioural Brain Research.* 417, 113571.

Hiura LC, Kelly AM, Ophir AG (2018) Age-specific and context-specific responses of the medial extended amygdala in the developing prairie vole. *Developmental Neurobiology*. 78, 1231-1245.

Hiura LC, Ophir AG (2018) Interactions between two stages of early life social experiences and sex shape nonapeptide receptor profiles. *Integrative Zoology*. 13, 745-760.

Kelly AM, Hiura LC, Ophir AG (2018) Rapid nonapeptide synthesis during a critical period of development in the prairie vole: Plasticity of the paraventricular nucleus of the hypothalamus. *Brain Structure and Function.* 223, 2547-2560.

Kelly AM, Saunders AG, Ophir AG (2018) Mechanistic substrates of a life history transition in male prairie voles: Developmental plasticity in affiliation and aggression corresponds to nonapeptide neuronal function. *Hormones and Behavior*. 99, 14-24.

Prounis GS, Thomas K, Ophir AG (2018) Developmental trajectories and influences of environmental complexity on oxytocin receptor and vasopressin 1a receptor expression in male and female prairie voles. *Journal of Comparative Neurology.* 526, 1820-1842.

Prounis GS, Ophir AG (2019) The impact of perinatal and juvenile social environments on the effects of chronic intranasal oxytocin in the prairie vole. *Frontiers in Behavioral Neuroscience*. 213, 206.
